# Amplitude-determined seizure-threshold, electric field modeling, and electroconvulsive therapy antidepressant and cognitive outcomes

**DOI:** 10.1038/s41386-023-01780-4

**Published:** 2024-01-11

**Authors:** Christopher C. Abbott, Jeremy Miller, Danielle Farrar, Miklos Argyelan, Megan Lloyd, Taylor Squillaci, Brian Kimbrell, Sephira Ryman, Thomas R. Jones, Joel Upston, Davin K. Quinn, Angel V. Peterchev, Erik Erhardt, Abhishek Datta, Shawn M. McClintock, Zhi-De Deng

**Affiliations:** 1 Department of Psychiatry, University of New Mexico, Albuquerque, NM USA; 2 Institute of Behavioral Science, Feinstein Institutes for Medical Research, Manhasset, NY USA; 3 Department of Psychiatry, The Zucker Hillside Hospital, Glen Oaks, NY USA; 4 Mind Research Network, Albuquerque, NM USA; 5 Department of Neurology, Albuquerque, NM USA; 6 Department of Psychiatry and Behavioral Sciences, Duke University School of Medicine, Durham, NC USA; 7 Department of Biomedical Engineering, Duke University, Durham, NC USA; 8 Department of Electrical and Computer Engineering, Duke University, Durham, NC USA; 9 Department of Neurosurgery, Duke University School of Medicine, Durham, NC USA; 10 Department of Mathematics and Statistics, University of New Mexico, Albuquerque, NM USA; 11 Soterix Medical, New York, NY USA; 12 Division of Psychology, Department of Psychiatry, UT Southwestern Medical Center, Dallas, TX USA; 13 Noninvasive Neuromodulation Unit, Experimental Therapeutics and Pathophysiology Branch, National Institute of Mental Health, National Institutes of Health, Bethesda, MD USA

**Keywords:** Translational research, Depression

## Abstract

Electroconvulsive therapy (ECT) pulse amplitude, which dictates the induced electric field (E-field) magnitude in the brain, is presently fixed at 800 or 900 milliamperes (mA) without clinical or scientific rationale. We have previously demonstrated that increased E-field strength improves ECT’s antidepressant effect but worsens cognitive outcomes. Amplitude-determined seizure titration may reduce the E-field variability relative to fixed amplitude ECT. In this investigation, we assessed the relationships among amplitude-determined seizure-threshold (ST_a_), E-field magnitude, and clinical outcomes in older adults (age range 50 to 80 years) with depression. Subjects received brain imaging, depression assessment, and neuropsychological assessment pre-, mid-, and post-ECT. ST_a_ was determined during the first treatment with a Soterix Medical 4×1 High Definition ECT Multi-channel Stimulation Interface (Investigation Device Exemption: G200123). Subsequent treatments were completed with right unilateral electrode placement (RUL) and 800 mA. We calculated *E*_brain_ defined as the 90th percentile of E-field magnitude in the whole brain for RUL electrode placement. Twenty-nine subjects were included in the final analyses. *E*_brain_ per unit electrode current, *E*_brain_/*I*, was associated with ST_a_. ST_a_ was associated with antidepressant outcomes at the mid-ECT assessment and bitemporal electrode placement switch. *E*_brain_/*I* was associated with changes in category fluency with a large effect size. The relationship between ST_a_ and *E*_brain_/*I* extends work from preclinical models and provides a validation step for ECT E-field modeling. ECT with individualized amplitude based on E-field modeling or ST_a_ has the potential to enhance neuroscience-based ECT parameter selection and improve clinical outcomes.

## Introduction

Despite the proven antidepressant efficacy of electroconvulsive therapy (ECT) for depressive episodes [1], procedure-related cognitive impairment remains a major concern [2]. Meta-analyses have demonstrated that the acute phase of ECT may transiently impair most cognitive domains including attention, verbal fluency, memory, and executive function [3, 4]. Although transient, the cognitive recovery time is highly variable and spans days to months [3]. Post-ECT cognitive impairment prolongs the period of functional impairment (e.g., unable to work) and delays recovery from severe depressive episodes [3, 5]. Consequences of ECT-cognitive impairment include reluctance to consider initiation of the procedure, hesitancy to restart ECT in the context of relapse, and perpetuation of ECT’s negative stigma [6–9]. Importantly, ECT-cognitive impairment may be a modifiable side effect [10].

Pulse amplitude, which dictates the induced electric field (E-field) strength in the brain, is presently fixed at 800 or 900 milliamperes (mA) without clinical or scientific rationale [11]. We recently completed a clinical trial with older adults (50–80 years) with depression who were randomized to different pulse amplitudes (600, 700, and 800 mA) with a focus on E-field and hippocampal volume change. The focus on older subjects captured age-related involutional changes that increased E-field variability with fixed amplitude ECT [12]. The results of that investigation demonstrated a trade-off between cognitive outcomes (stable in the 600 mA arm) and antidepressant outcomes (improved with the 700 and 800 mA arms) [13]. E-field strength was also variable within each arm and challenged the use of a fixed amplitude for older patients treated with ECT [14]. Like amplitude, increased E-field strength demonstrated a trade-off between cognitive safety (improved with a lower E-field) and antidepressant response via increased hippocampal volume change (improved with a higher E-field).

Since amplitude determines the E-field strength, amplitude-determined seizure titration may reduce the E-field variability relative to fixed amplitude ECT. Research in preclinical models demonstrated the feasibility of this approach and validated ECT E-field modeling [15, 16]. In this investigation, we performed an amplitude-determined seizure titration with right unilateral electrode placement (RUL) to identify the amplitude at which seizure activity was initiated (ST_a_) in older subjects (50–80 years) with depression. Subjects then received RUL with 800 mA amplitude at all subsequent treatments with a bitemporal (BT) contingency in the context of inadequate antidepressant response at the mid-point evaluation. Based on previous work, increased E-field strength appears to be related to increased right hippocampal volume change [14, 17]. We calculated E_brain_ as the 90th percentile of E-field magnitude in the whole brain for RUL electrode placement. Increased *E*_brain_ per unit applied electrode current (*E*_brain_/*I*)is associated with greater efficiency of the injected current to simulate the brain, for example due to a smaller skull diameter, thinner skull, or larger brain size. Therefore, increased *E*_brain_/*I* is expected to be associated with decreased ST_a_. Our primary hypotheses evaluated if 1) increased ST_a_ will be associated with decreased *E*_brain_/*I* and 2) increased ST_a_ will be associated with decreased right hippocampal volume change. In addition, we assessed the relationships of ST_a_, *E*_brain_/*I*, and right hippocampal volume change on antidepressant and cognitive outcomes.

## Methods

### Participants

The overall study design has been registered as a clinical trial (ClinicalTrials.gov Identifier: NCT04621786). The University of New Mexico Human Research Protections Office approved this investigation (20–601). All subjects provided written informed consent to the research protocol and study participation. Inclusion criteria consisted of the following: 1) major depressive disorder (MDD; with or without psychotic features) confirmed with two separate psychiatric evaluations [18]; 2) clinical indications for ECT with RUL electrode placement including treatment resistance or a need for a rapid and definitive response [19]; 3) right-handedness, and 4) age range between 50 and 80 years. Subjects remained on antidepressant medication treatment throughout the ECT series with antidepressant medication dose titrations permitted during the ECT series [20]. Exclusion criteria consisted of the following*:* 1) Defined neurological or neurodegenerative disorder (e.g., traumatic brain injury, epilepsy, Alzheimer’s disease); 2) other psychiatric conditions (e.g., schizophrenia, bipolar disorder) as the primary indication for ECT; 3) current drug or alcohol use disorder (except for nicotine); and 4) contraindications to MRI. See supplemental material for power analyses and sample size determination.

### Study protocol

Subjects received their baseline brain imaging, clinical, and neuropsychological assessment 24–48 h before the first ECT session (V1). Amplitude-determined seizure titration (detailed below) was completed during the first treatment with subsequent treatments completed with RUL and 800 mA. The ECT series continued thrice weekly until clinically determined endpoints, which included non-response, antidepressant plateau, or remission [19]. Subjects received their second assessment (V2) one day after the sixth ECT treatment and the final assessment (V3) within one week after the acute phase of treatment. The timing of the V2 assessment allows sufficient time to evaluate the effectiveness of RUL 800 mA and change to BT electrode placement if indicated to ensure that every patient receives an adequate ECT series. Each study visit included magnetic resonance imaging (MRI), clinical, and neuropsychological assessments. The anesthesiologist determined the appropriate dosage of general anesthetic (methohexital unless not available) and succinylcholine (depolarizing neuromuscular blocker). Anesthetic medications, electroencephalographic seizure duration, and maximum ictal heart rate were recorded for each treatment.

### Clinical assessments

The clinician-rated 30-item Inventory of Depressive Symptomatology (IDS-C) measured depression severity [21]. Each item is scored from 0–3 and summed for a total score between 0–84. The initial visit (V1) included the Maudsley Staging Method to measure antidepressant treatment resistance within the current depressive episode [22], ECT Appropriateness Scale to assess the indication for ECT [23], Medical History form to gauge overall medical burden, and Edinburgh Handedness Inventory to define handedness [24]. Additional characteristics of the current and past depressive episodes were also recorded during the initial visit: age of onset, age of first treatment, number of depressive episodes, and current depressive episode duration.

### Neuropsychological assessments

The Test of Premorbid Function (TOPF) estimated premorbid intellectual function for use as a covariate in cognitive analyses [25]. The Montreal Cognitive Assessment (MoCA, version 7.1), a measure of global cognitive function that is sensitive to gross neurocognitive abnormalities, screened for preexisting cognitive impairment [26, 27]. The Delis Kaplan Executive Function System (DKEFS) included Verbal Fluency (Letter and Category to measure phonemic and semantic fluency), Color-Word Interference (processing speed, inhibition, initiation, and cognitive flexibility), and the Tower Test (planning and problem-solving) [28–32]. The California Verbal Learning Test -3rd Edition (CVLT-3) measured verbal learning and memory [33]. The Dot Counting Test measured performance validity [34]. The Digit Span subtest from the Wechsler Adult Intelligence Scale-4th Edition (WAIS-IV) measured attention and working memory [35]. Our primary cognitive outcome was the DKEFS Verbal Fluency test (letter and category fluency) based on sensitivity to detect cognitive impairment with RUL [13].

### MRI acquisition

T1 data was collected with the following parameters: Repetition time (TR) = 2530 milliseconds (ms), echo time (TE) = 1.64, 3.5, 5.36, 7.22, 9.08 ms, Inversion time (TI) = 1200 ms, flip angle = 7.0°, slices = 192, field of view = 256, matrix 256 × 256, voxel size = 1.0 × 1.0 × 1.0 millimeter (mm) and total acquisition time 6:03 (minutes:seconds). T2 data was collected with the following parameters: TR = 2530 ms, TE = 474 ms, flip angle = 120.0°, slices = 192, field of view = 256, matrix 256 × 256, voxel size = 1.0 × 1.0 × 1.0 mm and total acquisition time = 5:09.

### Amplitude-determined seizure titration

Subjects received amplitude-determined seizure titration during the first treatment with RUL electrode placement [36]. The United States Food and Drug Administration (US FDA) approved the Soterix Medical 4 × 1 High Definition ECT Multi-channel Stimulation Interface for use in this investigation (Investigation Device Exemption: G200123). For the purposes of this investigation, we only used the amplitude reducer function of the interface. Subjects received stimulations starting with the lowest setting ~100 mA with ~70 mA increases at 30 s intervals until seizure activity was initiated (ST_a_). The delivered current was verified with an oscilloscope (RIGOL DS1074Z). EEG and right lower extremity motor activity confirmed seizure activity. Pulse width (1.0 milliseconds (ms)), pulse train duration (8 s), and frequency (20 hertz (Hz), 160 pulse pairs) were fixed for amplitude titration.

### Electroconvulsive therapy

Pulse width (1.0 ms), pulse train duration (8 s), and frequency (20 Hz, 160 pulse pairs) were fixed for the remaining 800 mA RUL treatments. By fixing the temporal stimulus parameters, the only difference between amplitude determined seizure titration and subsequent treatments was amplitude. Relatively low frequency (20 Hz) was used since it induces seizures more efficiently [37, 38]. Brief pulses (1.0 ms) maximize antidepressant efficacy of RUL ECT [39, 40]. If the RUL 800 mA treatments failed to demonstrate antidepressant improvement at V2 (<25% reduction from baseline IDS-C_30_ total score), subjects would then receive BT electrode placement, fixed 800 mA amplitude, 1.0 ms pulse width, and traditional fixed amplitude seizure titration based on step-wise increases in pulse train duration and frequency. Subsequent BT treatments were then delivered at two times the charge for the remainder of the ECT series.

### FreeSurfer segmentation

FreeSurfer 6.0 segmented the cortical and subcortical anatomy with a longitudinal pipeline [41–44]. We processed all the time points separately with the default FreeSurfer workflow and created an unbiased template from all the time points for each subject. Once this template was created, parcellations and segmentation were carried out at each time point initialized with common information from the within-subject template [42]. We calculated the percent change of the right hippocampus relative to the pre-treatment hippocampal volume.

### E-field modeling

The objective of E-field modeling in this study was to characterize the individual strength of the E-field delivered to the brain for a given electrode current amplitude. E-field modeling represents a single ECT pulse and does not include differences related to the temporal aspects of stimulation parameters (pulse width, train duration, and frequency), which were fixed for RUL ECT. The SimNIBS software (ver. 3.2.3 with headreco segmentation algorithm) was used to create a subject-specific, anatomically realistic volume conductor model [45]. The quasi-static approximation was assumed, which considers bioelectric currents in living tissues as stationary and resistive [46, 47]. The T1- and T2-weighted scans were segmented into biological tissues and converted to a tetrahedral head mesh using Gmsh, a three-dimensional finite element (FE) mesh generator. Unique conductivity values for each tissue type were based on previous research: cerebrospinal fluid: (1.654 siemens/meter (S/m)), vitreous bodies (0.50 S/m), scalp (0.465 S/m), gray matter (0.275 S/m), white matter (0.126 S/m), spongy bone (0.025 S/m), and compact bone (0.0008 S/m) [45]. ECT electrodes were added to the head mesh in a RUL configuration (C2 and FT8 based on the 10–20 system). SimNIBS then used a FE solver to calculate the electric potentials and electric fields that correspond to the stimulation throughout the head mesh. We calculated E_brain_ as the 90th percentile of E-field magnitude in the whole brain for RUL electrode placement to avoid the influence of tissue boundary effects that could bias the absolute maximum E-field values [16]. *E*_brain_/*I* is the E-field magnitude per unit stimulus current amplitude (in units of volts/meter (V/m) per milliampere (mA)). The *E*_brain_/*I* ratio can then be multiplied by the electrode current during amplitude-determined seizure or during the latter 800 mA treatments to determine the electric field strength with the applied amplitude. We focused on *E*_brain_/*I* as the locations of seizure generation, antidepressant response, and cognitive impairment are not known.

### Statistical analyses

We restricted the analysis to treatments completed with RUL electrode placement. We performed summary statistics on all clinical and demographic measures and assessed longitudinal change with paired t-tests. We used linear regressions to assess the relationships of 1) *E*_brain_/*I* on ST_a_; 2) ST_a_ on right hippocampal volume change; and 3) *E*_brain_/*I* on right hippocampal volume change. Covariates included age, sex, and treatment number (for right hippocampal volume change [48]) and the inclusion of interactions with Akaike Information Criterion. Regression diagnostics were consistent with model assumptions. We also assessed the relationships between ST_a_, *E*_brain_/*I*, and right hippocampal percent volume change with depression severity (percent change of IDS-C) and cognitive outcomes (change in DKEFS Letter and Category Fluency Summary Scores). Sex and TOPF standard score were included as covariates for cognitive outcomes (age was included in demographic-adjusted Scaled Scores). We present the results with right hippocampal volume change and clinical outcomes with *E*_brain_/*I* with the qualification that *E*_brain_800_ is proportional to *E*_brain_/*I*. We performed logistic regressions for significant antidepressant (electrode placement switch, responder/non-responder) and cognitive relationships (impaired/not impaired with dichotomization with <−3 scaled score for letter, category fluency outcomes [28]). If logistic regression demonstrated a relationship, we performed receiver operating characteristic curves to determine the sensitivity and specificity at the empirical cut-point. Finally, we compared the regional E-field (i.e., *E*_r-hippo_/*I*, *E*_r-amygdala_/*I*) strength for 166 cortical and subcortical FreeSurfer regions with antidepressant and cognitive outcomes.

## Results

### Clinical characteristics

**Fig. 1:**
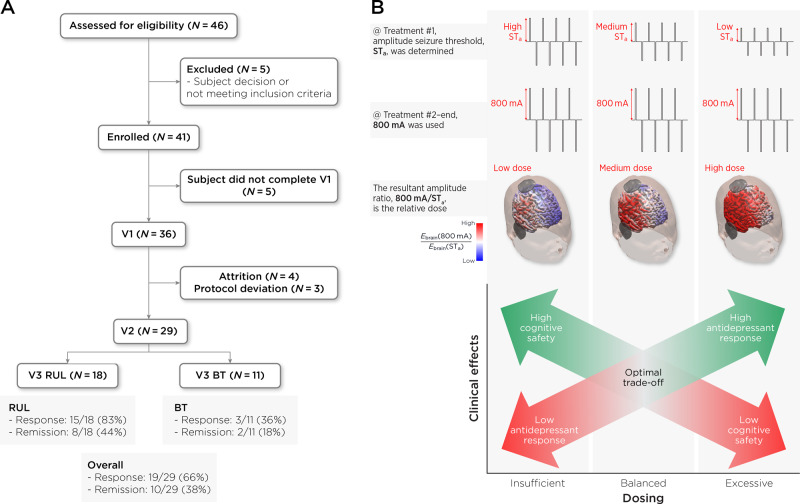
Subject flow and hypothesized relationships. **A** Subject flow and attrition based on pre- (V1), mid- (V2), and post-ECT (V3) time points. **B** Study protocol and hypotheses: The first treatment determined an amplitude seizure threshold (STa). Subsequent treatments were completed at 800 mA. The resultant amplitude ratio 800 mA/STa determines the relative dose of ECT. Low STa followed by 800 mA will result in a “high” dose and cognitive impairment. High STa followed by 800 mA will result in a “low” dose that is insufficient for antidepressant response.

Forty-one subjects enrolled in study protocol from March 2021 to September 2022 consistent with pre-defined enrollment goals. Thirty-two subjects completed baseline (V1) assessment and received amplitude-determined seizure threshold titration. Three subjects were excluded from final analysis based on the following protocol deviations: pulse width error, amplitude titration error, and non-protocol determined switch to BT electrode placement. Five subjects received propofol when methohexital was unavailable, and they were included in the final analyses of twenty-nine subjects (Fig. 1A). The demographic, clinical characteristics, antidepressant, and cognitive outcomes are summarized in Table 1. The average age and sex distribution of the study sample was 64.2 (+/−7.9 Standard deviation (SD)) years and 12 male/17 female.

**Table 1:** Clinical and demographics, neuropsychological results.

Clinical and demographic features	Pre-ECT mean (SD)	Post-ECT mean (SD)	t-Statistic (*p*-value)
Age +/− SD (years)	64.2 (7.9)		
Sex: Male/Female	12/17		
Race: White/Black/Asian	27/1/1		
Ethnicity: Non-Hispanic/Hispanic	24/5		
Single episode/recurrent	1/28		
Psychotic/non-psychotic	5/24		
Episode duration (months)	44.4 (59.0)		
Number of episodes	4.3 (3.0)		
Age of onset (years)	28.8 (17.1)		
Lifetime duration (years)	17.2 (16.2)		
ECT appropriateness scale	8.5 (1.4)		
Maudsley treatment failure	2.4 (1.3)		
Education^a^	5.8 (1.7)		
Antidepressants: SSRI/SNRI/TCA/NDRI/NaSSA/SMS^b^	7/12/3/2/2/3		
Antipsychotics/Mood Stabilizers/Benzodiazepines	14/0/10		
**Amplitude-determined seizure, Ebrain, seizure duration**			
Amplitude-determined seizure (milliampere)	312.3 (112.9)		
Ebrain (Volts/meter per milliampere)	0.15 (0.02)		
EEG seizure duration titration (seconds)	42.5 (38.8)		
EEG seizure duration 800 mA (seconds)	38.9 (18.8)		
RUL treatment number	8.1 (2.8)		
**Depression assessment**			
Inventory of depressive symptoms – clinician rated	48.5 (10.0)	27.3 (16.1)	6.6 (<0.001)
**Cognitive assessments**			
Montreal cognitive assessment	23.9 (3.8)		
Test of premorbid functioning standard score total	108.6 (13.9)		
Delis Kaplan executive function system			
Letter fluency scaled score	8.8 (3.8)	7.9 (4.0)	2.4 (0.03)
Category fluency scaled score	9.2 (4.6)	7.7 (3.9)	2.7 (0.01)
Category switching total switching accuracy scaled score	8.6 (4.1)	8.3 (3.8)	0.4 (0.66)
Color-word interference condition 2 scaled score	9.3 (3.1)	8.3 (3.8)	2.2 (0.04)
Color-word interference condition 3 scaled score	9.1 (3.1)	7.9 (4.3)	1.4 (0.18)
Tower total achievement scaled score	9.65 (3.0)	10.1 (3.0)	−0.8 (0.46)
California verbal learning test -3rd edition			
Trial 1 free recall correct standard score	4.4 (1.4)	10.7 (3.4)	−11.3 (<0.001)
Long delay free recall correct standard score	7.9 (3.4)	8.8 (3.6)	−1.7 (0.11)
Total recall correct sum of scaled scores	55.9 (20.0)	63.2 (19.6)	−2.5 (0.02)
Standard score summary – trials I – 4 correct sum of scaled scores	32.3 (12.3)	37.9 (10.9)	−3.1 (0.005)
Delayed recall correct sum of scaled scores	23.6 (9.3)	25.3 (10.1)	−1.2 (0.23)
Dot counting test			
Mean ungrouped time (seconds)	7.3 (2.0)	8.7 (3.1)	−2.6 (0.01)
Mean grouped time (seconds)	3.2 (2.6)	2.6 (1.2)	2.3 (0.03)
E-score	11.5 (3.6)	12.6 (4.0)	−1.4 (0.17)
Wechsler adult intelligence scale IV digit span scaled score	10.4 (3.3)	10.0 (1.9)	0.8 (0.39)

^a^Education, 1 = grade 6 or less, 2 = grade 7–12, 3 = graduated high school, 4 = part college or university, 5 = graduated 2-year college, 6 = graduated 4-year college, 7 = part graduate or professional school, 8 = completed graduate or professional school.

^b^*SSRI* select serotonin reuptake inhibitor, *SNRI* serotonin-norepinephrine reuptake inhibitor, *TCA* tricyclic antidepressant, *NDRI* norepinephrine-dopamine reuptake inhibitor, *NaSSA* noradrenergic and specific serotonergic antidepressant, *SMS* serotonin modulator and stimulator.

### Amplitude-determined seizure (ST_a_), E_brain_, and right hippocampal volume change

**Fig. 2:**
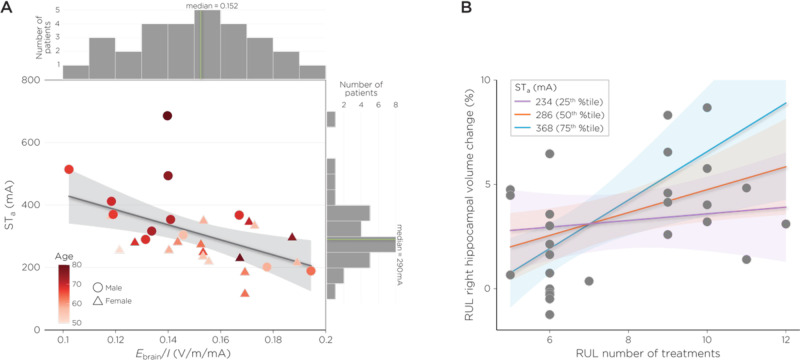
*E*_brain_/*I*, ST_a_, and right hippocampal volume relationships. **A** *E*_brain_/*I* was associated with amplitude-determined seizure titration (ST_a_, eta-squared = 0.26). Older subjects had higher ST_a_ (color bar, eta-squared = 0.21). **B** ST_a_ and treatment number interaction was associated with right hippocampal volume change (eta-squared = 0.24). Lower ST_a_ had an earlier right hippocampal volume increase that plateaued with higher treatment number.

ST_a_ (312.34 mA, +/−113.07 SD, range: 120–686) and *E*_brain_/*I* (0.15 V/m/mA, +/−0.02, range: 0.10–0.19) had considerable range (Fig. 2A). The average *E*_brain_ for amplitude-determined seizure (*E*_brain_ST_) was 45.6 (+/−14.31) V/m. *E*_brain_/*I* was associated with ST_a_ (β = −2074.16, *t*_25_ = −2.95, *p* = 0.007, eta-squared = 0.26). Age (β = 5.61, *t*_25_ = 2.55, *p* = 0.02, eta-squared = 0.21) but not sex (β = −45.47, *t*_25_ = −1.26, *p* = 0.22, eta-squared = 0.06) explained additional variance associated with ST_a_. ST_a_ and treatment number interaction was associated with right hippocampal volume change (β = 0.000075, *t*_21_ = 2.24, *p* = 0.04, eta-squared = 0.24) such that lower ST_a_ had an earlier right hippocampal volume increase that plateaued with higher treatment number (Fig. 2B). *E*_brain_/*I* and treatment number interaction was not associated with right hippocampal volume change (β = −0.21, *t*_21_ = −1.88, *p* = 0.07, eta-squared = 0.17).

### Antidepressant outcomes

**Fig. 3:**
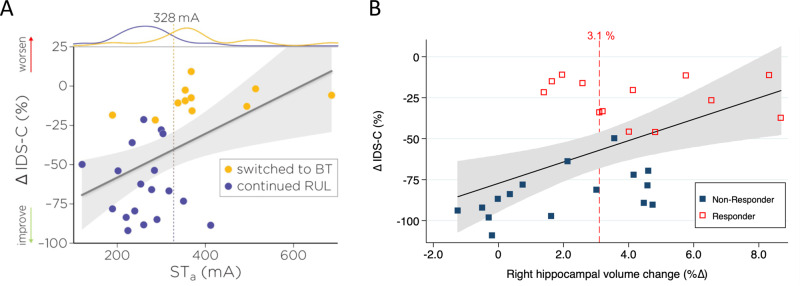
ST_a_ and right hippocampal volume change were associated with antidepressant outcomes. **A** Higher ST_a_ was associated with reduced change in depression severity at the mid-ECT assessment (eta-squared = 0.20) and differentiated bitemporal electrode placement switch. The dashed line is the cut-point (328 mA) associated with bitemporal electrode placement switch (area under the ROC curve: 0.85). **B** Right hippocampal volume change was associated with antidepressant outcomes (eta-squared = 0.20) and differentiated RUL response criteria (3.1% volume change, dashed red vertical line).

Increased ST_a_ was associated with poor antidepressant outcomes at V2 (β = −0.0012, *t*_25_ = −2.48, *p* = 0.02, eta-squared = 0.20) but not at the V3 assessment (β = −0.0012, *t*_25_ = −2.04, *p* = 0.05, eta-squared = 0.14) (Fig. 3A). ST_a_ did not differentiate RUL response/non-response outcomes (β = −0.013, z_25_ = −1.93, *p* = 0.05) but did differentiate BT electrode placement switch (β = 0.021, z_25_ = 2.33, *p* = 0.02, empirical cut point = 328 mA with sensitivity = 0.82 and specificity = 0.89, area under the curve (AUC) = 0.85). *E*_brain_/*I* was not associated with antidepressant outcomes (β = 0.016, *t*_25_ = 0.01, *p* = 0.99, eta-squared <0.01). Increased right hippocampal volume change was associated with improved antidepressant outcomes (β = 5.27, *t*_23_ = 2.37, *p* = 0.03, eta-squared = 0.20). Right hippocampal volume change differentiated RUL response/non-response outcomes (β = 45.64, z_23_ = 1.98, *p* = 0.048, empirical cut point = 3.1% with sensitivity = 0.79 and specificity = 0.67, AUC = 0.73) and BT switch at V2 (β = −61.96, z_23_ = −2.06, *p* = 0.04, empirical cut point = 4.3% with sensitivity = 0.18 and specificity = 0.76, AUC = 0.20) (Fig. 3B). Whole brain analysis comparing regional electric field strength and antidepressant outcomes did not reveal any relationships (uncorrected *p* > 0.05).

### Cognitive outcomes

**Fig. 4:**
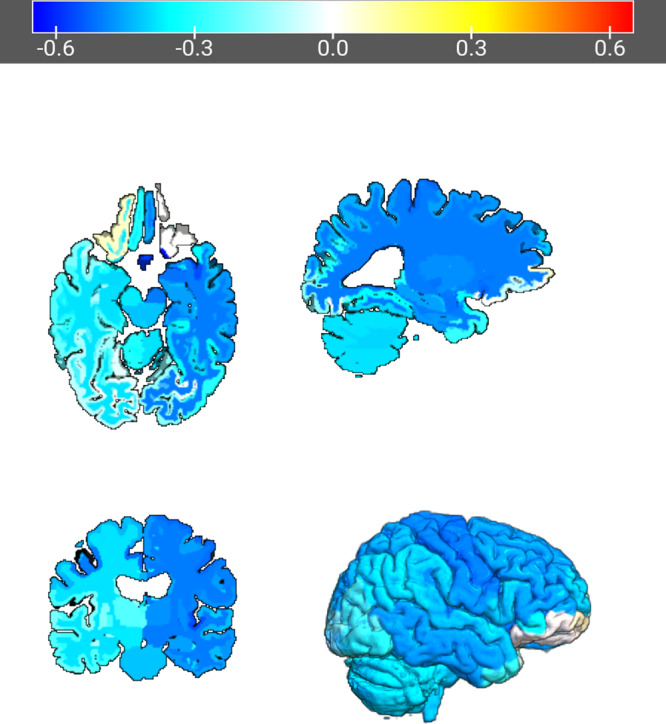
Increased *E*_brain_/*I* was associated with impaired DKEFS Category Fluency performance (eta-squared = 0.29). Whole brain analysis revealed widespread right hemisphere relationships with increased regional E-field strength and impaired cognitive performance (54 of 166 regions with *p*_FDR_ < 0.05).

ST_a_ was not associated with changes in letter (*β* = 0.0025, *t*_24_ = 0.60, *p* = 0.55, eta-squared = 0.01) or category fluency (*β* = 0.0090, *t*_24_ = 1.61, *p* = 0.12, eta-squared = 0.10). *E*_brain_/*I* was not associated with changes in letter fluency (*β* = −28.28, *t*_24_ = −1.62, *p* = 0.12, eta-squared = 0.10) but was associated with changes in category fluency with a large effect size (*β* = −68.74, *t*_24_ = −3.15, *p* = 0.004, eta-squared = 0.29). *E*_brain_/*I* did not differentiate dichotomous category fluency outcomes (*β* = 33.75, z_24_ = 1.72, *p* = 0.09). Right hippocampal volume change was not associated with letter (*β* = −33.46, *t*_23_ = −1.85, *p* = 0.08, eta-squared = 0.13) or category fluency change (*β* = −47.26, *t*_23_ = −1.84, *p* = 0.08, eta-squared = 0.13). In an exploratory analysis, 16/29 subjects had impaired longitudinal performance with either letter or category fluency. *E*_brain_/*I* differentiated dichotomous combined letter and category fluency outcomes (*β* = 57.15, z_23_ = 2.15, *p* = 0.03, empirical cut point = 0.15 V/m/mA or 119 V/m at 800 mA with sensitivity = 0.69 and specificity = 0.69, AUC = 0.69). Whole brain analysis comparing regional electric field strength and category fluency outcomes revealed widespread right hemisphere associations (Fig. 4, see also Supplemental Material). From the 54 cortical parcellations that demonstrated a relationship with category fluency (pFDR < 0.05), 44 parcellations were from the right hemisphere. Right subcortical segmentations included the right pallidum, amygdala, nucleus accumbens, diencephalon, putamen, thalamus and hippocampus.

## Discussion

This investigation used a unique design with amplitude-determined seizure titration at the first treatment followed by fixed 800 mA for subsequent treatments. Pulse number (20 Hz frequency and 8 s pulse train duration) was held constant at 160 pulse pairs for the ST_a_ titration and the 800 mA treatments. Both ST_a_ and *E*_brain_/*I* had a wide range, which challenges the long-standing use of fixed amplitude ECT. ST_a_ increased with age, which is consistent with the observations made by Liberson when “brief stimulus therapy” was first experimented in the 1940s [49]. The relationship between ST_a_ and *E*_brain_/*I* extends work from preclinical models [15, 16] and provides a validation step for ECT E-field modeling. Despite their relationship, ST_a_ and *E*_brain_/*I* had different relationships with antidepressant and cognitive outcomes. The antidepressant and cognitive outcomes in relation to ST_a_ may be understood as a ratio between ST_a_ and subsequent 800 mA treatments (800 mA/STa) (Fig. 1B). Lower ratios (e.g., 800 mA/686 mA) may be inadequate to achieve the necessary “suprathreshold” dosing for antidepressant response. Higher ST_a_ (and hence lower 800 mA/ST_a_ ratio) was associated with the BT switch at V2 as determined by <25% change from baseline depression severity. Higher *E*_brain_/*I* was associated with worse cognitive outcomes as measured by longitudinal change in category fluency. *E*_brain_/*I* at the mid-point of the distribution (0.15 V/m/mA) differentiated cognitive impairment with 800 mA amplitude. ST_a_ and treatment number interaction was related to right hippocampal volume change, and right hippocampal volume change was associated with antidepressant outcomes (bitemporal switch and RUL response). In the following sections, we provide context for these findings, strengths and limitations of this approach, and potential implications for the practice of ECT dosing.

### Antidepressant outcomes

Our antidepressant results demonstrated improved efficacy with “suprathreshold” treatments. In current clinical practice, suprathreshold treatments are defined in the context of pulse number (i.e., pulse train duration and frequency). The minimum number of pulses for a fixed amplitude and pulse width determines the seizure threshold. The suprathreshold multiplier (typically six-times seizure threshold for RUL ECT) determines the individualized pulse number necessary for antidepressant efficacy [50]. In contrast, the suprathreshold specifier can also be applied to the stimulus current amplitude. Our findings indicate that for ECT with 800 mA fixed amplitude to be effective, the inflection point for ST_a_ is approximately 330 mA, corresponding to fixed amplitude/ST_a_ ratio of 800 mA/330 mA or ~2.5. Higher ST_a_ (>330 mA) is associated with inadequate antidepressant response with subsequent treatments completed at 800 mA resulting in a protocol-determined switch to BT electrode placement. When the ST_a_ is greater than 330 mA, increased amplitude (>800 mA) may improve antidepressant efficacy. Alternatively, an electrode placement switch from RUL to bitemporal may also provide the necessary suprathreshold dose for antidepressant efficacy in the context of high ST_a_.

Previous research with E-field strength and antidepressant outcomes has been mixed [14, 17, 51]. In this current study sample, *E*_brain_/*I* was unrelated to antidepressant outcomes. We also did not replicate the previously identified relationship between right hippocampal E-field strength (*E*_hippo_/*I*) and right hippocampal volume change [14]. Differences between these two investigations include the focus on RUL (previous investigation included RUL and BT) and 800 mA (previous investigation included 600 and 700 mA). Despite not demonstrating the *E*_brain_/*I* and hippocampal volume relationship, right hippocampal volume change was associated with antidepressant outcome including response criteria and bitemporal electrode placement switch. Larger investigations are necessary to disentangle the effect of *E*_brain_/*I* from ECT treatment number and other parameters (i.e., pulse width, stimulation time) on hippocampal volume change and to explore potential moderating effects of structural and functional changes between *E*_brain_/*I* and antidepressant outcomes. In contrast, ST_a_ may capture additional information such as cortical excitability not included in *E*_brain_/*I* that strengthens the relationships to antidepressant outcomes [52] or age-related changes in conductivity (i.e., white matter disease) not presently included in E-field modeling approaches [53].

### Cognitive outcomes

The DKEFS Category and Letter Fluency tests were sensitive to RUL-mediated changes in cognitive performance. Higher *E*_brain_/*I* was associated with worse category fluency performance. In contrast, ST_a_ was unrelated with cognitive outcomes. The strong association between *E*_brain_/*I* and cognitive outcomes replicates our previous work and adds support for the role of *E*_brain_/*I* in ECT dosing [14, 54]. In this sample, an *E*_brain_/*I* of 0.15 V/m/mA was the maximal associated with stable cognitive performance with traditional fixed amplitude 800 mA dosing. When *E*_brain_/*I* is greater than 0.15 V/m/mA (120 V/m at 800 mA), decreased amplitude (<800 mA) may reduce cognitive risk. The widespread right hemisphere associations between *E*_brain_/*I* and cognitive outcomes did not identify a specific anatomic “anti-target” amenable to changes in electric field geometry to improve the focality of treatment to prevent cognitive impairment. In contrast, an individualized stimulus amplitude determined prior to treatment initiation has the potential to improve cognitive outcomes.

### Limitations

We discuss the following limitations to assist with result interpretation and future directions. First, this investigation had a relatively small sample for an ECT-imaging investigation. Second, we focused exclusively on RUL electrode placement. The results may not generalize to other electrode placements (bifrontal, bitemporal). Third, our amplitude titration included coarse approximation of 70 mA steps. A more granular and accurate step size (i.e., 25 mA) may improve the relationships with *E*_brain_/*I* and ST_a_ but would have resulted in more stimulation steps. Fourth, the E-field modeling employs the quasi-static approximation [55], models a single pulse of current, and does not model white matter age-related changes [56] or non-linear tissue impedances [57]. We used fixed temporal ECT parameters (pulse width, frequency, and duration) to focus exclusively on amplitude and reduce confounds from effects related to the E-field temporal characteristics. E-field modeling is rapidly advancing with improved segmentation algorithms for non-brain tissues but is only available for research applications at this time [58]. Further work is necessary to validate E-field modeling for clinical applications across the adult lifespan. Larger ECT E-field samples are also necessary to establish the optimal *E*_brain_. Finally, our primary hypotheses regarding the relationships between *E*_brain_/*I* and ST_a_ were pre-registered, but the analyses included repeated analyses for *E*_brain_ and ST_a_ for clinical outcomes without corrections for multiple comparisons.

### Future directions and implications for ECT dosing

Currently, most ECT clinicians implement a trial-and-error approach to parameter selection, initially favoring reduced cognitive risk (RUL and ultrabrief pulse width) before advancing to other parameters with increased cognitive risk (bitemporal and brief pulse width) [19]. This trial-and-error approach may still expose patients to increased cognitive risk with RUL (i.e., patient has high *E*_brain_/*I*) and may miss the optimal dose for antidepressant response without cognitive risk. The fixed current amplitude approach fails to consider individual anatomic variability resulting in variable brain E-field and sub-optimal clinical outcomes. An individualized amplitude that is sufficient for antidepressant effect with greater cognitive safety can be determined prior to treatment initiation. Individualized current amplitude can also eliminate the trial-and-error methods of ECT parameter selection thus reducing the overall number of treatments of the ECT series and expediting antidepressant response.

The first approach to individualized amplitude uses ST_a_ during the first treatment with a suprathreshold multiplier for subsequent treatments. Based on our data, the highest ST_a_ associated with antidepressant response at 800 mA was 330 mA, indicating 2.5× ST_a_ as an appropriate suprathreshold current multiplier. Similar to pulse number titrations, the first treatment would be sub-therapeutic with no expectations of antidepressant efficacy. The second approach uses pre-treatment E-field modeling. The optimal individualized current amplitude can be determined by dividing the optimal E-field strength by the individual *E*_brain_/*I*. The optimal E-field strength is sufficient to induce an antidepressant effect without cognitive impairment. Results to date suggest that the relationship between *E*_brain_ and cognitive outcome may provide an upper threshold for optimal dosing (preliminary results suggest that this value is between 110–120 V/m). E-field informed ECT would eliminate the dose finding approach to the first treatment and start with therapeutic stimulation, but would require MRI acquisition and processing. Yet another potential approach is to carry out a non-convulsive motor threshold titration through the ECT electrodes, and leverage its correlation with ST_a_ and *E*_brain_/*I* observed in preclinical studies [15, 59]. After amplitude individualization, further research should seek to identify optimal frequency and number of pulses and determine if they vary across subjects. Any of these approaches to ECT current individualization would require commercial device development for fine amplitude adjustments (~1 mA) and lower starting amplitude (100 mA), although such devices have been available for experimental studies [60]. Regardless of approach, individualized amplitude has the potential to advance neuroscience-based ECT dosing strategies and optimize both antidepressant and cognitive outcomes.

### Supplementary information

Supplemental information
