# Supplementary material for: Amplitude-determined seizure-threshold, electric field modeling, and electroconvulsive therapy antidepressant and cognitive outcomes
Source: Neuropsychopharmacology. 2024 Jan 11;49(4):640–8. doi: 10.1038/s41386-023-01780-4 (PMC10876627; doi:10.1038/s41386-023-01780-4)
Supplement: Supplementary file 1 — Supplemental information [file 41386_2023_1780_MOESM1_ESM.docx]

Supplemental Table 1. Freesurfer regions associated with change in DKEFS Verbal Fluency Category Fluency Summary Scores

|  | Freesurfer ROI | Freesurfer Region | Mean electric field (V/m) | r | p | p_adj |
| --- | --- | --- | --- | --- | --- | --- |
| 1 | 52 | Right-Pallidum | 96.840 | -0.534 | 0.003 | 0.040 |
| 2 | 54 | Right-Amygdala | 57.315 | -0.512 | 0.005 | 0.040 |
| 3 | 58 | Right-Accumbens-area | 94.402 | -0.520 | 0.004 | 0.040 |
| 4 | 60 | Right-VentralDC | 85.148 | -0.502 | 0.005 | 0.040 |
| 5 | 11108 | ctx-lh-G_frontal_inf-Triangular_part | 70.794 | -0.520 | 0.004 | 0.040 |
| 6 | 11127 | ctx-lh-G_precuneus | 40.109 | -0.539 | 0.003 | 0.040 |
| 7 | 11130 | ctx-lh-G_subcentral | 51.934 | -0.505 | 0.005 | 0.040 |
| 8 | 11146 | ctx-lh-S_central_insula | 46.462 | -0.503 | 0.005 | 0.040 |
| 9 | 11147 | ctx_lh_S_cingul-Marginalis | 53.000 | -0.532 | 0.003 | 0.040 |
| 10 | 11157 | ctx-lh-S_frontomarginal | 40.382 | -0.538 | 0.003 | 0.040 |
| 11 | 11170 | ctx-lh-S_pericallosal | 49.845 | -0.503 | 0.005 | 0.040 |
| 12 | 12103 | ctx_rh_G_and_S_paracentral | 68.738 | -0.501 | 0.006 | 0.040 |
| 13 | 12108 | ctx_rh_G_and_S_cingul-Mid-Post | 76.515 | -0.494 | 0.006 | 0.040 |
| 14 | 12118 | ctx_rh_G_insular_short | 79.976 | -0.499 | 0.006 | 0.040 |
| 15 | 12128 | ctx_rh_G_postcentral | 107.073 | -0.496 | 0.006 | 0.040 |
| 16 | 12129 | ctx_rh_G_precentral | 113.111 | -0.524 | 0.004 | 0.040 |
| 17 | 12130 | ctx_rh_G_precuneus | 55.947 | -0.526 | 0.003 | 0.040 |
| 18 | 12136 | ctx_rh_G_temp_sup-Plan_tempo | 113.324 | -0.500 | 0.006 | 0.040 |
| 19 | 12141 | ctx_rh_Lat_Fis-post | 95.870 | -0.494 | 0.006 | 0.040 |
| 20 | 12146 | ctx_rh_S_central | 108.954 | -0.516 | 0.004 | 0.040 |
| 21 | 12147 | ctx_rh_S_cingul-Marginalis | 64.848 | -0.511 | 0.005 | 0.040 |
| 22 | 12148 | ctx_rh_S_circular_insula_ant | 89.203 | -0.530 | 0.003 | 0.040 |
| 23 | 12149 | ctx_rh_S_circular_insula_inf | 96.061 | -0.558 | 0.002 | 0.040 |
| 24 | 12150 | ctx_rh_S_circular_insula_sup | 102.096 | -0.498 | 0.006 | 0.040 |
| 25 | 12151 | ctx_rh_S_collat_transv_ant | 92.405 | -0.497 | 0.006 | 0.040 |
| 26 | 12153 | ctx_rh_S_front_inf | 102.701 | -0.502 | 0.006 | 0.040 |
| 27 | 12164 | ctx_rh_S_orbital_med-olfact | 76.536 | -0.631 | 0.000 | 0.040 |
| 28 | 12169 | ctx_rh_S_precentral-inf-part | 121.553 | -0.499 | 0.006 | 0.040 |
| 29 | 12154 | ctx_rh_S_front_middle | 79.819 | -0.490 | 0.007 | 0.041 |
| 30 | 12117 | ctx_rh_G_Ins_lg_and_S_cent_ins | 81.875 | -0.488 | 0.007 | 0.042 |
| 31 | 12163 | ctx_rh_S_orbital_lateral | 92.744 | -0.485 | 0.008 | 0.043 |
| 32 | 51 | Right-Putamen | 85.426 | -0.483 | 0.008 | 0.043 |
| 33 | 12109 | ctx_rh_G_cingul-Post-dorsal | 74.675 | -0.482 | 0.008 | 0.043 |
| 34 | 49 | Right-Thalamus-Proper | 68.688 | -0.478 | 0.009 | 0.044 |
| 35 | 53 | Right-Hippocampus | 60.358 | -0.459 | 0.012 | 0.044 |
| 36 | 11107 | ctx_lh_G_and_S_cingul-Mid-Ant | 64.426 | -0.460 | 0.012 | 0.044 |
| 37 | 11109 | ctx_lh_G_cingul-Post-dorsal | 71.816 | -0.462 | 0.012 | 0.044 |
| 38 | 11172 | ctx_lh_S_subparietal | 56.110 | -0.467 | 0.011 | 0.044 |
| 39 | 12104 | ctx_rh_G_and_S_subcentral | 123.438 | -0.474 | 0.009 | 0.044 |
| 40 | 12107 | ctx_rh_G_and_S_cingul-Mid-Ant | 69.597 | -0.462 | 0.012 | 0.044 |
| 41 | 12114 | ctx_rh_G_front_inf-Triangul | 111.870 | -0.469 | 0.010 | 0.044 |
| 42 | 12115 | ctx_rh_G_front_middle | 102.403 | -0.467 | 0.011 | 0.044 |
| 43 | 12116 | ctx_rh_G_front_sup | 65.705 | -0.472 | 0.010 | 0.044 |
| 44 | 12123 | ctx_rh_G_oc-temp_med-Parahip | 73.608 | -0.474 | 0.009 | 0.044 |
| 45 | 12137 | ctx_rh_G_temporal_inf | 89.024 | -0.459 | 0.012 | 0.044 |
| 46 | 12138 | ctx_rh_G_temporal_middle | 121.295 | -0.471 | 0.010 | 0.044 |
| 47 | 12140 | ctx_rh_Lat_Fis-ant-Vertical | 108.533 | -0.463 | 0.012 | 0.044 |
| 48 | 12155 | ctx_rh_S_front_sup | 84.840 | -0.472 | 0.010 | 0.044 |
| 49 | 12168 | ctx_rh_S_postcentral | 93.416 | -0.469 | 0.010 | 0.044 |
| 50 | 12170 | ctx_rh_S_precentral-sup-part | 94.742 | -0.462 | 0.012 | 0.044 |
| 51 | 12172 | ctx_rh_S_subparietal | 62.196 | -0.460 | 0.012 | 0.044 |
| 52 | 12131 | ctx_rh_G_rectus | 62.600 | -0.451 | 0.014 | 0.048 |
| 53 | 12127 | ctx_rh_G_parietal_sup | 58.660 | -0.448 | 0.015 | 0.049 |
| 54 | 12120 | ctx_rh_G_occipital_sup | 49.840 | -0.447 | 0.015 | 0.050 |
| 55 | 50 | Right-Caudate | 76.762 | -0.445 | 0.016 | 0.050 |
| 56 | 11116 | ctx_lh_G_front_sup | 52.931 | -0.443 | 0.016 | 0.050 |
| 57 | 11168 | ctx_lh_S_postcentral | 41.084 | -0.443 | 0.016 | 0.050 |
| 58 | 12174 | ctx_rh_S_temporal_sup | 96.689 | -0.442 | 0.016 | 0.050 |
| 59 | 12157 | ctx_rh_S_intrapariet_and_P_trans | 68.625 | -0.439 | 0.017 | 0.051 |
| 60 | 12133 | ctx_rh_G_temp_sup-G_T_transv | 95.756 | -0.435 | 0.018 | 0.053 |
| 61 | 12173 | ctx_rh_S_temporal_inf | 96.967 | -0.431 | 0.020 | 0.056 |
| 62 | 11129 | ctx_lh_G_precentral | 43.921 | -0.430 | 0.020 | 0.056 |
| 63 | 11128 | ctx_lh_G_postcentral | 39.652 | -0.426 | 0.021 | 0.057 |
| 64 | 12134 | ctx_rh_G_temp_sup-Lateral | 143.109 | -0.427 | 0.021 | 0.057 |
| 65 | 11103 | ctx_lh_G_and_S_paracentral | 50.990 | -0.421 | 0.023 | 0.060 |
| 66 | 12132 | ctx_rh_G_subcallosal | 66.092 | -0.421 | 0.023 | 0.060 |
| 67 | 11156 | ctx_lh_S_interm_prim-Jensen | 40.604 | -0.412 | 0.026 | 0.065 |
| 68 | 12105 | ctx_rh_G_and_S_transv_frontopol | 59.084 | -0.411 | 0.027 | 0.065 |
| 69 | 12112 | ctx_rh_G_oc-temp_med-Lingual | 107.323 | -0.413 | 0.026 | 0.065 |
| 70 | 12126 | ctx_rh_G_pariet_inf-Supramar | 120.837 | -0.414 | 0.026 | 0.065 |
| 71 | 12175 | ctx_rh_S_temporal_transverse | 108.511 | -0.413 | 0.026 | 0.065 |
| 72 | 11155 | ctx_lh_S_front_sup | 48.553 | -0.410 | 0.027 | 0.066 |
| 73 | 12166 | ctx_rh_S_parieto_occipital | 56.244 | -0.402 | 0.031 | 0.073 |
| 74 | 18 | Left-Amygdala | 32.284 | -0.396 | 0.033 | 0.074 |
| 75 | 12121 | ctx_rh_G_oc-temp_lat-fusifor | 55.597 | -0.400 | 0.032 | 0.074 |
| 76 | 12125 | ctx_rh_G_pariet_inf-Angular | 83.506 | -0.397 | 0.033 | 0.074 |
| 77 | 12139 | ctx_rh_Lat_Fis-ant-Horizont | 89.216 | -0.397 | 0.033 | 0.074 |
| 78 | 11164 | ctx_lh_S_orbital_med-olfact | 34.428 | -0.393 | 0.035 | 0.076 |
| 79 | 17 | Left-Hippocampus | 34.583 | -0.388 | 0.037 | 0.081 |
| 80 | 47 | Right-Cerebellum-Cortex | 43.183 | -0.387 | 0.038 | 0.081 |
| 81 | 28 | Left-VentralDC | 60.958 | -0.373 | 0.046 | 0.096 |
| 82 | 11120 | ctx_lh_G_occipital_sup | 38.357 | -0.373 | 0.047 | 0.096 |
| 83 | 12161 | ctx_rh_S_oc-temp_lat | 61.171 | -0.358 | 0.056 | 0.114 |
| 84 | 11132 | ctx_lh_G_subcallosal | 53.444 | -0.353 | 0.061 | 0.121 |
| 85 | 12162 | ctx_rh_S_oc-temp_med_and_Lingual | 58.538 | -0.352 | 0.061 | 0.121 |
| 86 | 12135 | ctx_rh_G_temp_sup-Plan_polar | 106.641 | -0.350 | 0.063 | 0.123 |
| 87 | 11125 | ctx_lh_G_pariet_inf-Angular | 38.041 | -0.349 | 0.063 | 0.123 |
| 88 | 26 | Left-Accumbens-area | 66.333 | -0.347 | 0.065 | 0.124 |
| 89 | 12113 | ctx_rh_G_front_inf-Orbital | 93.239 | -0.348 | 0.065 | 0.124 |
| 90 | 11115 | ctx_lh_G_front_middle | 40.966 | -0.344 | 0.068 | 0.128 |
| 91 | 12111 | ctx_rh_G_cuneus | 49.506 | -0.341 | 0.070 | 0.131 |
| 92 | 12156 | ctx_rh_S_interm_prim-Jensen | 107.776 | -0.341 | 0.070 | 0.131 |
| 93 | 11166 | ctx_lh_S_parieto_occipital | 45.819 | -0.336 | 0.074 | 0.134 |
| 94 | 12167 | ctx_rh_S_pericallosal | 76.577 | -0.332 | 0.078 | 0.140 |
| 95 | 11159 | ctx_lh_S_oc_sup_and_transversal | 37.905 | -0.309 | 0.102 | 0.177 |
| 96 | 12159 | ctx_rh_S_oc_sup_and_transversal | 55.109 | -0.310 | 0.102 | 0.177 |
| 97 | 12171 | ctx_rh_S_suborbital | 64.049 | -0.308 | 0.104 | 0.178 |
| 98 | 8 | Left-Cerebellum-Cortex | 36.900 | -0.307 | 0.105 | 0.178 |
| 99 | 11131 | ctx_lh_G_rectus | 33.712 | -0.302 | 0.111 | 0.184 |
| 100 | 11141 | ctx_lh_Lat_Fis-post | 34.906 | -0.302 | 0.111 | 0.184 |
| 101 | 11169 | ctx_lh_S_precentral-inf-part | 44.729 | -0.302 | 0.112 | 0.184 |
| 102 | 12102 | ctx_rh_G_and_S_occipital_inf | 49.317 | -0.297 | 0.117 | 0.191 |
| 103 | 11173 | ctx_lh_S_temporal_inf | 29.498 | -0.293 | 0.122 | 0.196 |
| 104 | 12106 | ctx_rh_G_and_S_cingul-Ant | 56.279 | -0.294 | 0.122 | 0.196 |
| 105 | 11167 | ctx_lh_S_pericallosal | 71.423 | -0.291 | 0.126 | 0.200 |
| 106 | 11126 | ctx_lh_G_pariet_inf-Supramar | 38.463 | -0.288 | 0.129 | 0.203 |
| 107 | 11123 | ctx_lh_G_oc-temp_med-Parahip | 28.600 | -0.285 | 0.134 | 0.209 |
| 108 | 12144 | ctx_rh_Pole_temporal | 90.128 | -0.277 | 0.146 | 0.225 |
| 109 | 11175 | ctx_lh_S_temporal_transverse | 35.365 | -0.263 | 0.168 | 0.255 |
| 110 | 11104 | ctx_lh_G_and_S_subcentral | 39.143 | -0.257 | 0.178 | 0.263 |
| 111 | 11174 | ctx_lh_S_temporal_sup | 33.283 | -0.258 | 0.177 | 0.263 |
| 112 | 12119 | ctx_rh_G_occipital_middle | 60.342 | -0.258 | 0.176 | 0.263 |
| 113 | 13 | Left-Pallidum | 53.218 | -0.249 | 0.193 | 0.279 |
| 114 | 11138 | ctx_lh_G_temporal_middle | 31.830 | -0.243 | 0.204 | 0.290 |
| 115 | 11171 | ctx_lh_S_suborbital | 36.108 | -0.243 | 0.204 | 0.290 |
| 116 | 11151 | ctx_lh_S_collat_transv_ant | 26.027 | -0.241 | 0.209 | 0.295 |
| 117 | 11136 | ctx_lh_G_temp_sup-Plan_tempo | 34.276 | -0.240 | 0.211 | 0.296 |
| 118 | 10 | Left-Thalamus-Proper | 45.564 | -0.238 | 0.213 | 0.297 |
| 119 | 12145 | ctx_rh_S_calcarine | 44.845 | -0.237 | 0.216 | 0.299 |
| 120 | 11162 | ctx_lh_S_oc-temp_med_and_Lingual | 33.695 | -0.236 | 0.219 | 0.300 |
| 121 | 11133 | ctx_lh_G_temp_sup-G_T_transv | 31.946 | -0.228 | 0.235 | 0.318 |
| 122 | 11140 | ctx_lh_Lat_Fis-ant-Vertical | 39.990 | -0.214 | 0.266 | 0.356 |
| 123 | 11160 | ctx_lh_S_occipital_ant | 36.016 | -0.213 | 0.266 | 0.356 |
| 124 | 11119 | ctx_lh_G_occipital_middle | 36.554 | -0.211 | 0.271 | 0.359 |
| 125 | 12160 | ctx_rh_S_occipital_ant | 60.654 | -0.210 | 0.275 | 0.362 |
| 126 | 11137 | ctx_lh_G_temporal_inf | 28.122 | -0.208 | 0.280 | 0.366 |
| 127 | 11110 | ctx_lh_G_cingul-Post-ventral | 46.813 | -0.203 | 0.292 | 0.380 |
| 128 | 11150 | ctx_lh_S_circular_insula_sup | 34.758 | -0.201 | 0.295 | 0.381 |
| 129 | 12110 | ctx_rh_G_cingul-Post-ventral | 49.003 | -0.193 | 0.317 | 0.406 |
| 130 | 11106 | ctx_lh_G_and_S_cingul-Ant | 45.239 | -0.185 | 0.337 | 0.424 |
| 131 | 11153 | ctx_lh_S_front_inf | 37.570 | -0.185 | 0.337 | 0.424 |
| 132 | 11111 | ctx_lh_G_cuneus | 44.980 | -0.183 | 0.342 | 0.425 |
| 133 | 11134 | ctx_lh_G_temp_sup-Lateral | 32.829 | -0.184 | 0.341 | 0.425 |
| 134 | 12 | Left-Putamen | 41.413 | -0.180 | 0.350 | 0.427 |
| 135 | 11154 | ctx_lh_S_front_middle | 37.873 | -0.180 | 0.350 | 0.427 |
| 136 | 12158 | ctx_rh_S_oc_middle_and_Lunatus | 50.041 | -0.180 | 0.351 | 0.427 |
| 137 | 11149 | ctx_lh_S_circular_insula_inf | 34.311 | -0.177 | 0.358 | 0.434 |
| 138 | 11 | Left-Caudate | 36.000 | -0.169 | 0.382 | 0.459 |
| 139 | 12143 | ctx_rh_Pole_occipital | 40.534 | -0.166 | 0.390 | 0.466 |
| 140 | 11148 | ctx_lh_S_circular_insula_ant | 34.418 | -0.161 | 0.403 | 0.479 |
| 141 | 11105 | ctx_lh_G_and_S_transv_frontopol | 32.196 | -0.159 | 0.411 | 0.482 |
| 142 | 12122 | ctx_rh_G_oc-temp_med-Lingual | 40.808 | -0.157 | 0.415 | 0.482 |
| 143 | 11158 | ctx_lh_S_oc_middle_and_Lunatus | 33.869 | -0.156 | 0.418 | 0.482 |
| 144 | 11117 | ctx_lh_G_Ins_lg_and_S_cent_ins | 33.063 | -0.153 | 0.429 | 0.492 |
| 145 | 11163 | ctx_lh_S_orbital_lateral | 29.585 | -0.148 | 0.443 | 0.505 |
| 146 | 11118 | ctx_lh_G_insular_short | 31.198 | -0.140 | 0.470 | 0.530 |
| 147 | 11121 | ctx_lh_G_oc-temp_lat-fusifor | 26.632 | -0.137 | 0.480 | 0.538 |
| 148 | 11112 | ctx_lh_G_front_inf-Opercular | 37.757 | -0.135 | 0.484 | 0.540 |
| 149 | 11161 | ctx_lh_S_oc-temp_lat | 31.389 | -0.119 | 0.538 | 0.593 |
| 150 | 11114 | ctx_lh_G_front_inf-Triangul | 34.000 | -0.105 | 0.587 | 0.640 |
| 151 | 11135 | ctx_lh_G_temp_sup-Plan_polar | 29.700 | -0.101 | 0.603 | 0.653 |
| 152 | 11113 | ctx_lh_G_front_inf-Orbital | 29.102 | -0.099 | 0.608 | 0.655 |
| 153 | 11102 | ctx_lh_G_and_S_occipital_inf | 32.322 | -0.094 | 0.627 | 0.672 |
| 154 | 11145 | ctx_lh_S_calcarine | 36.411 | -0.085 | 0.661 | 0.705 |
| 155 | 12165 | ctx_rh_S_orbital-H_Shaped | 90.587 | -0.083 | 0.670 | 0.710 |
| 156 | 11143 | ctx_lh_Pole_occipital | 32.508 | -0.078 | 0.688 | 0.725 |
| 157 | 11124 | ctx_lh_G_orbital | 32.887 | 0.076 | 0.695 | 0.728 |
| 158 | 11144 | ctx_lh_Pole_temporal | 23.276 | -0.065 | 0.738 | 0.770 |
| 159 | 12101 | ctx_rh_G_and_S_frontomargin | 76.521 | 0.059 | 0.762 | 0.790 |
| 160 | 11101 | ctx_lh_G_and_S_frontomargin | 33.819 | 0.045 | 0.815 | 0.841 |
| 161 | 11122 | ctx_lh_G_oc-temp_med-Lingual | 31.431 | -0.044 | 0.821 | 0.842 |
| 162 | 11165 | ctx_lh_S_orbital-H_Shaped | 33.839 | -0.038 | 0.846 | 0.864 |
| 163 | 12152 | ctx_rh_S_collat_transv_post | 42.599 | -0.030 | 0.879 | 0.893 |
| 164 | 11139 | ctx_lh_Lat_Fis-ant-Horizont | 27.550 | -0.013 | 0.948 | 0.958 |
| 165 | 11152 | ctx_lh_S_collat_transv_post | 30.700 | 0.010 | 0.960 | 0.965 |
| 166 | 12124 | ctx_rh_G_orbital | 93.019 | 0.004 | 0.984 | 0.984 |

*Power analyses to determine sample size*

Hypothesis 1: Amplitude-determined seizure titration (ST_a_) will have a negative correlation with *E*_brain_/*I*. This is a replication of the results from the non-human primate data [1].

Power calculation: The observed correlation between ST_a_  and *I*/*E*_brain_ is *r* = 0.84 for non-human primates [1], and we expect that the more conservative Cohen’s “large” effect size *r* = 0.5 is a realistic lower bound for our population, thus a sample size of n = 29 provides 80% power at a 0.05 / 2 = 0.025 significance level.

Hypothesis 2: The ratio of amplitude titration to fixed amplitude ECT will demonstrate a linear relationship with treatment-responsive changes in hippocampal neuroplasticity.

Power calculation for H2: Based on our preliminary data, the relationship of E_brain_/I on percent hippocampal volume change (%Vol_Scaled_) has a correlation of *r* = −0.62 with an effect size f2 = 0.624, well above the Cohen's "large" effect size of f2 = 0.35. We will assume that the relationship between %Vol_Scaled_ will remain consistent with Cohen's "large" effect size. Then a sample size of n = 36 provides 80% power at a 0.05 significance level.

**References**

1 Lee WH, Lisanby SH, Laine AF, Peterchev AV. Minimum Electric Field Exposure for Seizure Induction with Electroconvulsive Therapy and Magnetic Seizure Therapy. Neuropsychopharmacology. 2017;42(6):1192-200.
